# Supplementary material for: Insights into Thai and Foreign Hemp Seed Oil and Extracts’ GC/MS Data Re-Analysis Through Learning Algorithms and Anti-Aging Properties
Source: Foods. 2025 Oct 31;14(21):3739. doi: 10.3390/foods14213739 (PMC12607487; doi:10.3390/foods14213739)
Supplement: Supplementary file 1 [file foods-14-03739-s001.zip › foods-3912109-supplementary.pdf]

Supplementary file

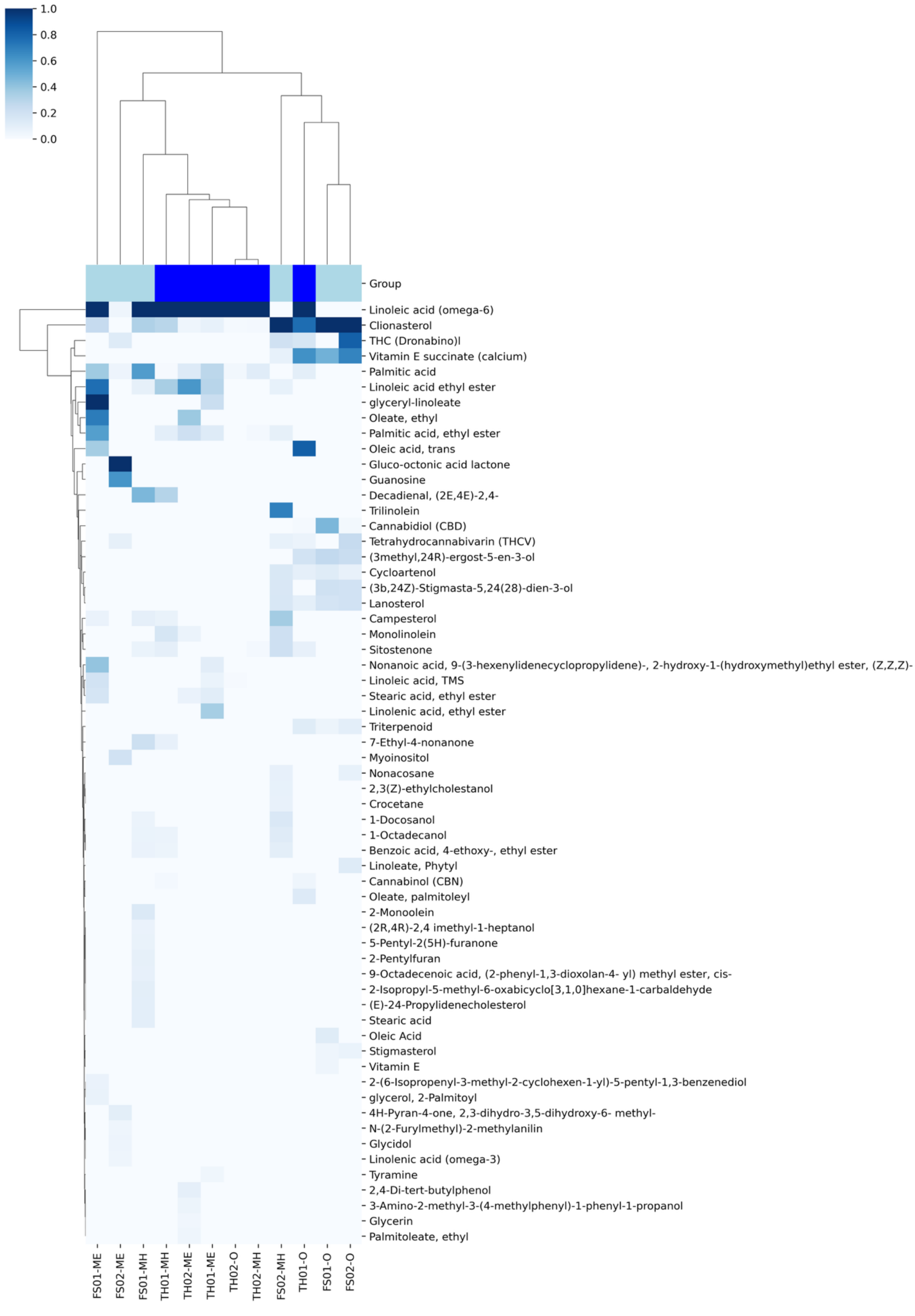

Figure S1. Hierarchy cluster map of Thai and foreign hemp cultivars' GC-MS data using % detection of each sample (in blue heatmap). On top of each heatmap, darker blue represents the Thai hemp cultivar's extracts, while lighter blue represents the foreign hemp cultivar's extracts. At the bottom of the heatmap, each cultivar extract is named in each column. There are twelve column names in total. FH is a foreign hemp cultivar (FH01 and FH02), and TH is a Thai hemp cultivar (TH01 and TH02). O stands for oil extract. ME is for hemp meat (hemp seed residue after oil extraction) ethanolic extract, and MH represents hemp meat hexane extract. This information is obtained from the authors' previous report.

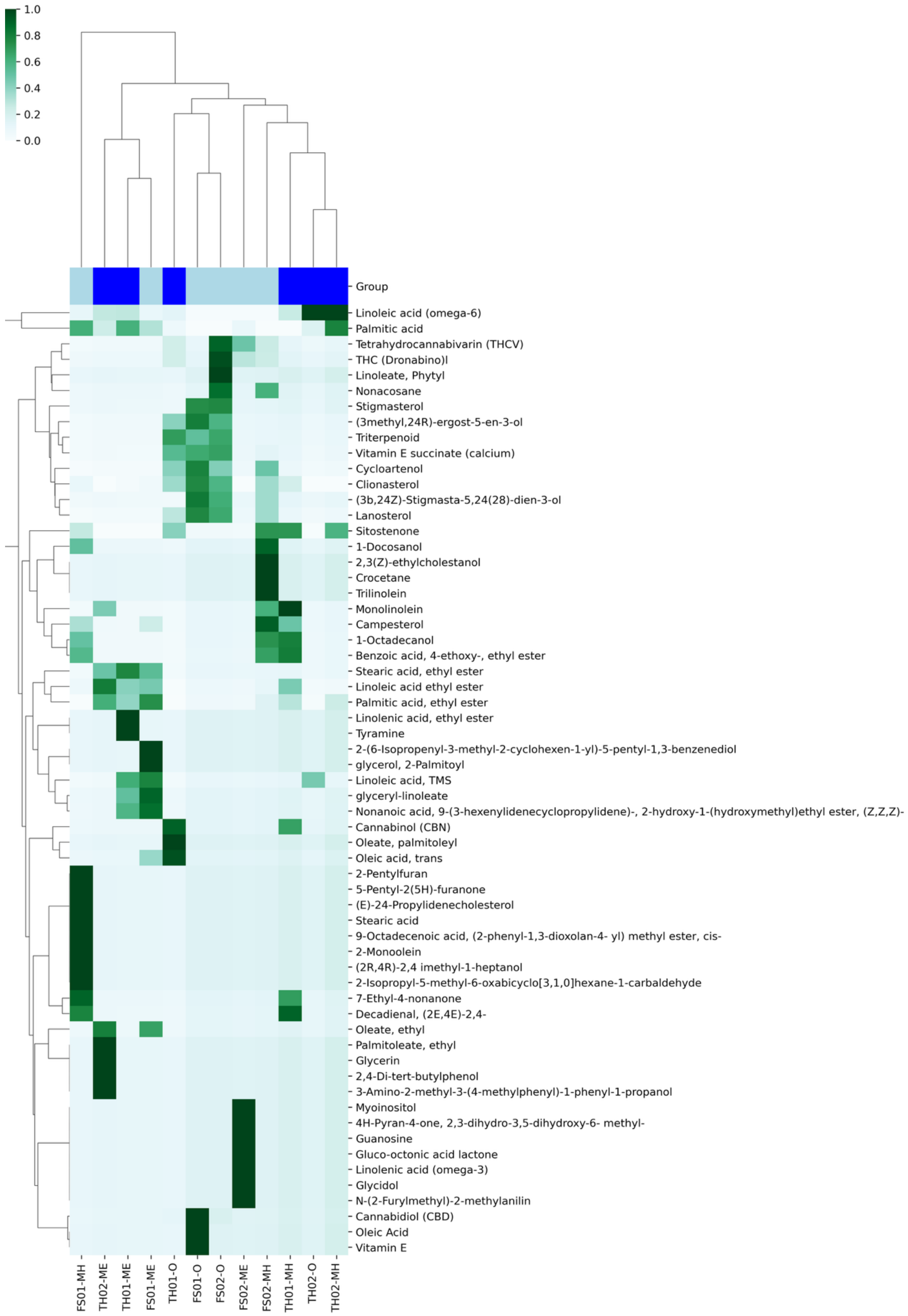

Figure S2. Hierarchy cluster map of Thai and foreign hemp cultivars' GC-MS data using normalized data with z-score (in green heatmap). On top of each heatmap, darker blue represents the Thai hemp cultivar's extracts, while lighter blue represents the foreign

hemp cultivar's extracts. At the bottom of the heatmap, each cultivar extract is named in each column. There are twelve column names in total. FH is a foreign hemp cultivar (FH01 and FH02), and TH is a Thai hemp cultivar (TH01 and TH02). O stands for oil extract. ME is for hemp meat (hemp seed residue after oil extraction) ethanolic extract, and MH represents hemp meat hexane extract. This information is obtained from the authors' previous report.

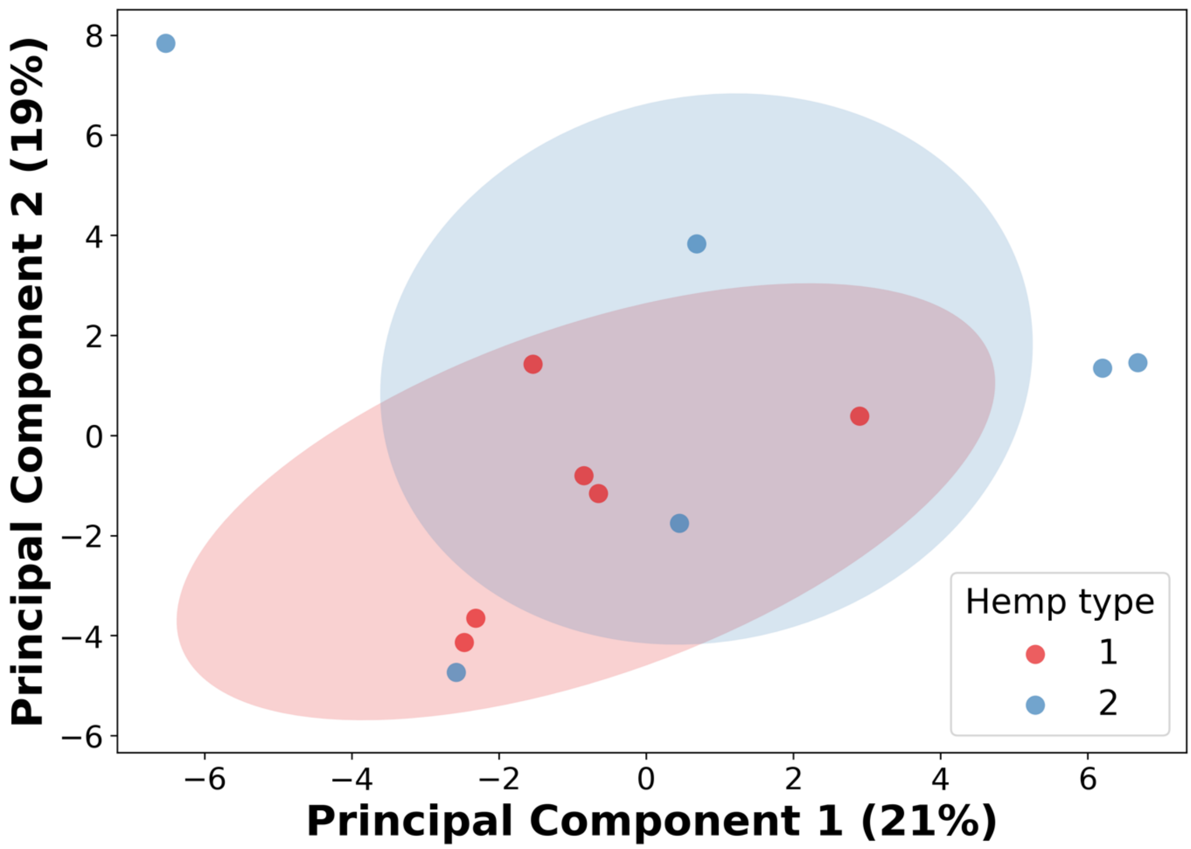

Figure S3. Principal component analysis (PCA) of complete GC-MS relative abundance data, sixty-one metabolic features. Hemp type 1 (red dots) represents hemp extracts from Thai cultivars, while hemp type 2 (blue dots) indicates hemp extracts from foreign cultivars.

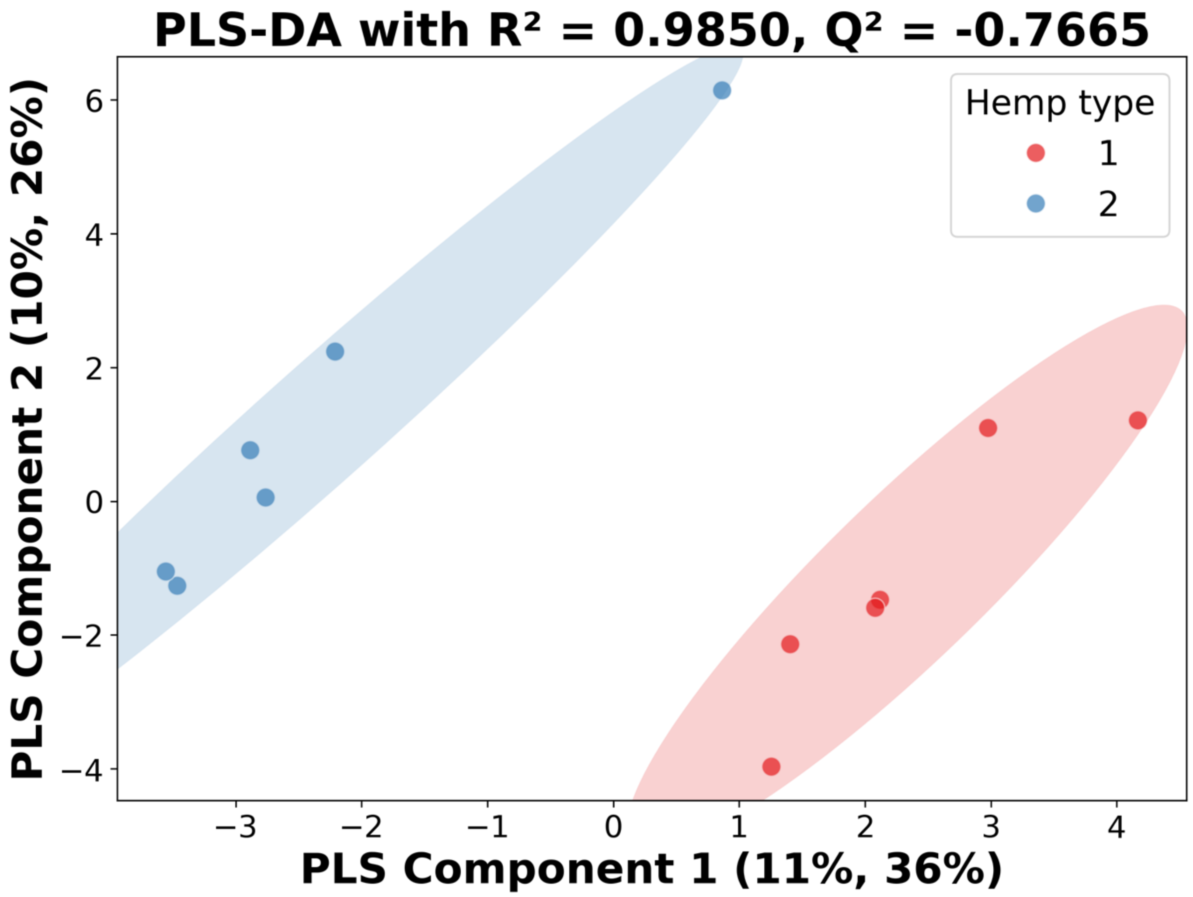

Figure S4. Partial least squares discriminant analysis (PLS-DA) of complete GC-MS relative abundance data, sixty-one metabolic features. Hemp type 1 (red dots) represents hemp extracts from Thai cultivars, while hemp type 2 (blue dots) indicates hemp extracts from foreign cultivars.

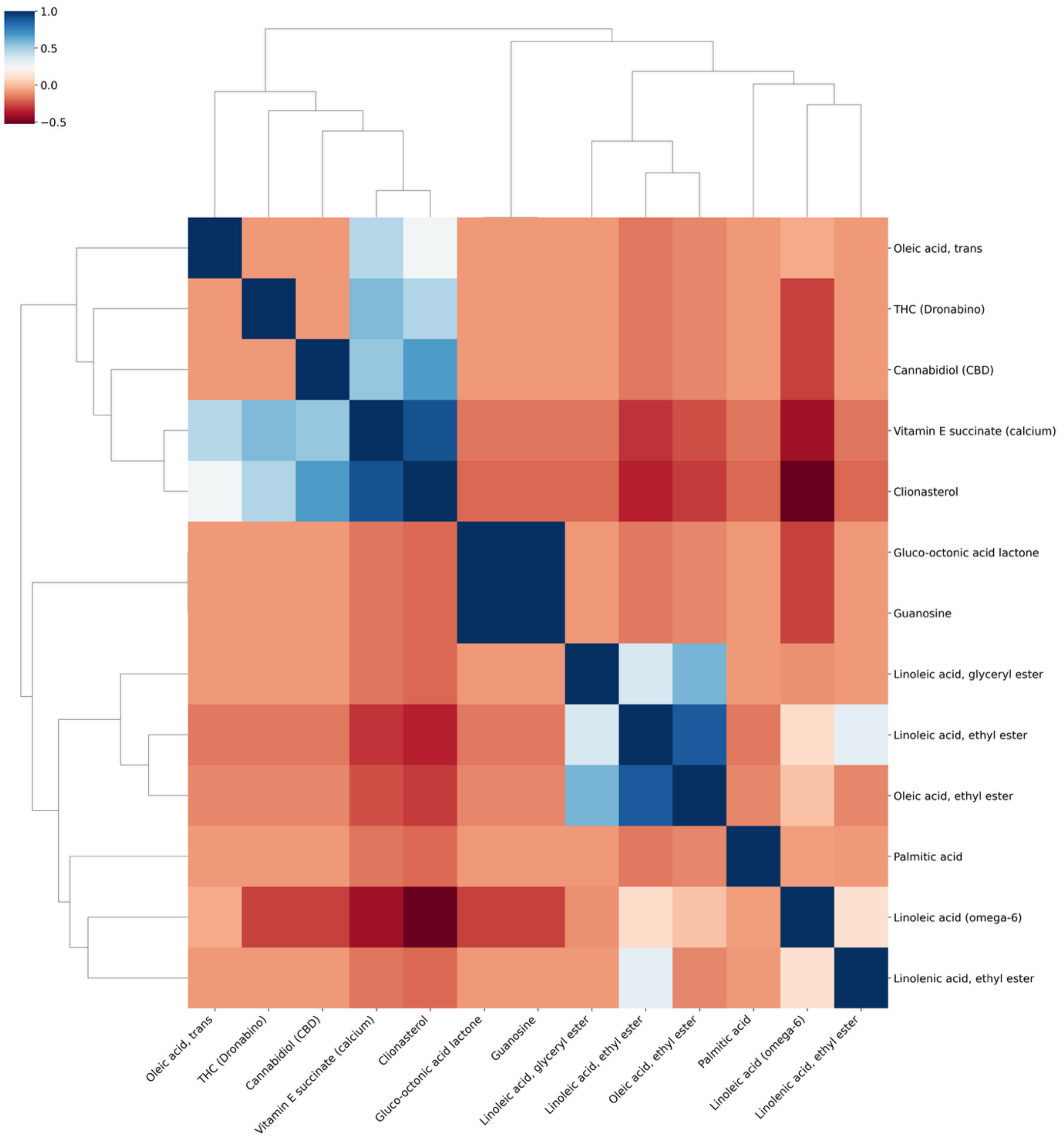

Figure S5. Pearson's correlation matrix of thirteen remaining metabolic features after the 10%vGC-MS relative abundance cutoff data.

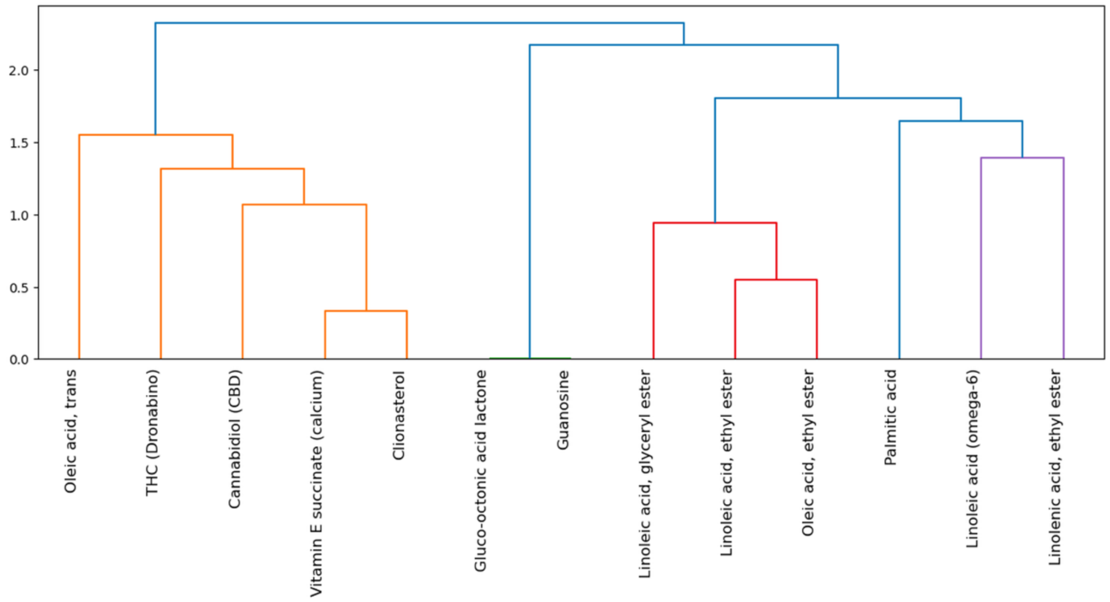

Figure S6. The obtained cluster from Pearson's correlation matrix. Five colors (orange, green, red, blue, and purple) represent four clusters of correlated metabolic features.

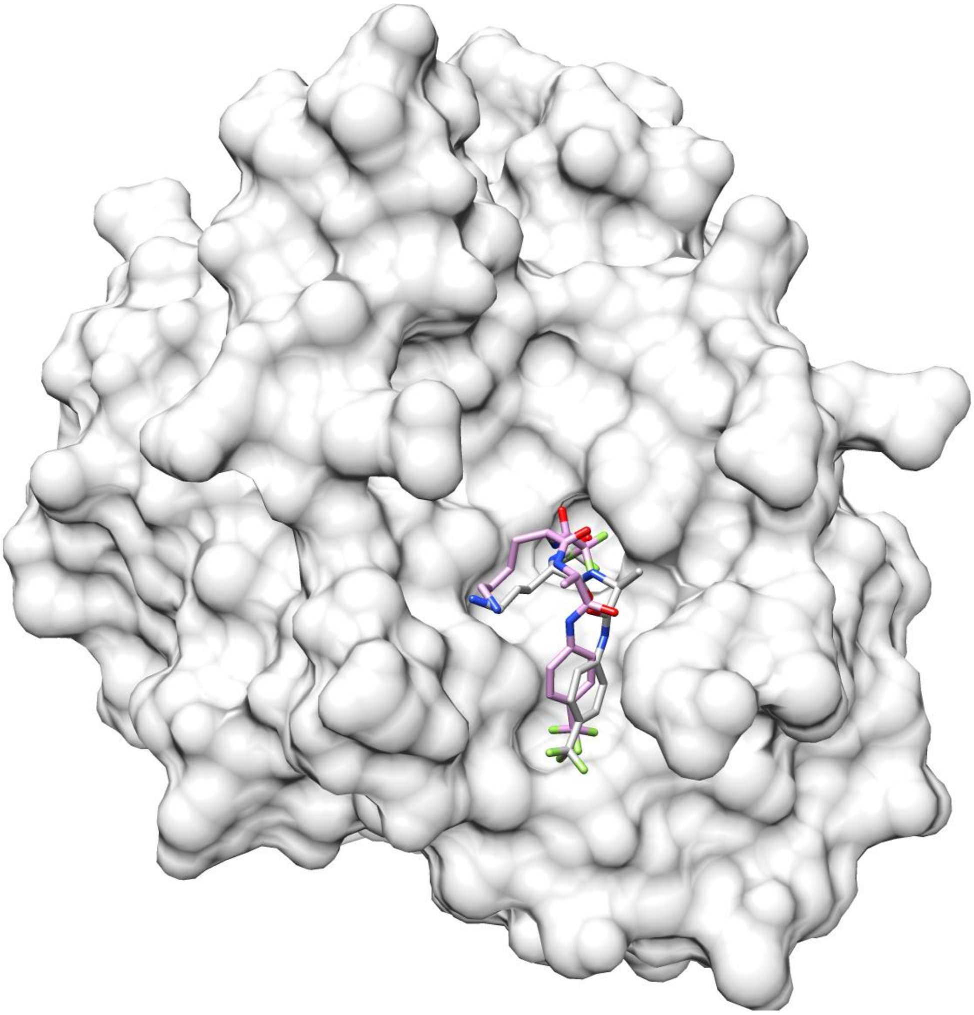

Figure S7. Docking validation of native ligand (grey color) and redocked ligand (pink color) on porcine elastase (PDB ID: 2EST). The redocking validation's root mean square deviation (RMSD) value is 2.661 Å, within the acceptance range of 3 Å.

Table S1. The thirteen remaining metabolic features after introducing a 10% abundance GC-MS data cutoff.

Type	Vitamin E succinate (calcium)	Clionasterol	Gluco-octonic acid lactone	Guanosine	THC (Dronabino)	Cannabidiol (CBD)	Linoleic acid (omega-6)	Linoleic acid, ethyl ester	Linoleic acid, glyceryl ester	Linolenic acid, ethyl ester	Oleic acid, ethyl ester	Oleic acid, trans	Palmitic acid
TH01-O	12,63	15,35	0	0	0	0	20,09	0	0	0	0	16,42	0
TH01-MH	0	0	0	0	0	0	22,93	0	0	0	0	0	0
TH01-ME	0	0	0	0	0	0	35,13	10,36	0	12,27	0	0	0
TH02-O	0	0	0	0	0	0	86,53	0	0	0	0	0	0
TH02-MH	0	0	0	0	0	0	66,24	0	0	0	0	0	0
TH02-ME	0	0	0	0	0	0	34,08	20,61	0	0	13,02	0	0
FS01-O	14,16	29,07	0	0	0	13,39	0	0	0	0	0	0	0
FS01-MH	0	0	0	0	0	0	17,63	0	0	0	0	0	10,21
FS01-ME	0	0	0	0	0	0	15,04	11,49	15,12	0	10,72	0	0
FS02-O	15,1	22,32	0	0	18,2	0	0	0	0	0	0	0	0
FS02-MH	0	13,42	0	0	0	0	0	0	0	0	0	0	0
FS02-ME	0	0	30,39	18,58	0	0	0	0	0	0	0	0	0
